# Non-invasive neuromodulation in reducing the risk of falls and fear of falling in community-dwelling older adults: systematic review

**DOI:** 10.3389/fnagi.2023.1301790

**Published:** 2024-03-05

**Authors:** Guilherme Augusto Santos Bueno, Arthur Dutra do Bomfim, Lorrane Freitas Campos, Anabela Correia Martins, Raquel Brito Elmescany, Marina Morato Stival, Silvana Schwerz Funghetto, Ruth Losada de Menezes

**Affiliations:** ^1^Department of Medicine, Centro Universitário Euro Americano, Brasilia, Brazil; ^2^Postgraduate Program in Health Sciences and Technologies, University of Brasilía, Brasilia, Brazil; ^3^LabinSaúde, Coimbra Health School Polytechnic of Coimbra, (ESTeSC-IPC), Coimbra, Portugal; ^4^Postgraduate Program in Health Sciences, Federal University of Goiás, Goiânia, Brazil

**Keywords:** transcranial magnetic stimulation, transcranial direct current stimulation, aging, fear of falling, fall accident

## Abstract

**Introduction:**

Neuromodulation is a non-invasive technique that allows for the modulation of cortical excitability and can produce changes in neuronal plasticity. Its application has recently been associated with the improvement of the motor pattern in older adults individuals with sequelae from neurological conditions.

**Objective:**

To highlight the effects of non-invasive neuromodulation on the risk of falls and fear of falling in community-dwelling older adults.

**Methods:**

Systematic review conducted in accordance with the items of the Cochrane Handbook for Systematic Reviews of Interventions. Searches were carried out in electronic databases: CENTRAL, Clinical Trials, LILACS, PEDro, PubMed, Web of Science, between 13/06/2020 and 20/09/2023, including all indexed texts without language and publication date restrictions, randomized controlled clinical trials, which presented as their main outcome non-invasive neuromodulation for reducing the fear of falling and risk of falls in the older adults, regardless of gender.

**Results:**

An extensive search identified 9 eligible studies for qualitative synthesis from 8,168 potential articles. Rigorous filtering through automated tools, title/abstract screening, and full-text evaluation ensured a focused and relevant selection for further analysis. Most studies (80%) used transcranial direct current electrical stimulation as an intervention, over the motor cortex or cerebellum area, with anodal current and monopolar electrode placement. The intensity ranged from 1.2 mA to 2 mA, with a duration of 20 min (80%). The profile of the research participants was predominantly individuals over 65 years old (80%), with a high risk of falls (60%) and a minority reporting a fear of falling (40%). The outcomes were favorable for the use of neuromodulation for the risk of falls in the older adults, through improvements in static and dynamic balance.

**Conclusion:**

The results may have limited applicability to direct outcomes related to the risk of falls, in addition to evidence regarding the difference or lack thereof in applicability between genders, fallers and non-fallers, as well as older adults individuals with low and high fear of falling.

**Systematic review registration:**

The protocol for this review was registered in the International Prospective Register of Systematic Reviews (PROSPERO) to obtain the identification of ongoing research (ID: 222429).

## 1 Introduction

A fall is defined as an event where a person unintentionally comes to rest on the ground or another lower level, excluding cases caused by a blow, loss of consciousness, sudden paralysis, or epilepsy (Tkacheva et al., 2023). Falls can result in serious health problems, including injuries, high medical costs, and a negative impact on quality of life (Topka et al., 2020). Falls have a significant impact on the quality of life of patients, potentially leading to institutionalization and dependence on caregivers (Lysyy, 2020). Fall prevention is crucial in reducing the risk of falls, especially among older individuals. Falls are a major health issue for older people, with a high incidence of hip fractures.

Falls among older adults have significant repercussions, including serious injuries, negative impacts on physical function, mobility, psychological well-being, independence, and the potential need for long-term care (Maita et al., 2023). They can lead to decreased independence, increased risk of morbidity and mortality, and greater dependency and disability (Hamieh et al., 2023). Injuries from falls are associated with disability, loss of independence, and increased mortality among older adults. Falls can also result in fractures, which can be complicated by spinal infections and pacemaker lead infections, further increasing morbidity and mortality (Topka et al., 2020). As a common reason for emergency department visits with potentially disastrous outcomes, fall prevention strategies are essential.

Various neurobiological factors are associated with falls, although their individual impact may vary. Balance control depends on both the peripheral and central nervous systems. The vestibular system, part of the peripheral system, plays a crucial role in maintaining postural balance and spatial orientation in response to environmental changes (Yoo and Mihaila, 2022). Vestibular feedback control is fundamental for dynamic stability during human locomotion (Schniepp et al., 2017). Additionally, the cerebellum, a vital component of the brain, regulates motor movement and balance control. It coordinates gait, maintains posture, and controls muscle tone and voluntary activity, though it cannot initiate muscle contraction (Yoo and Mihaila, 2022).

The frontal and temporal lobes of the brain also play a significant role in conscious balance control. The frontal lobe is crucial for controlling movement, maintaining balance, and executing locomotion. It utilizes cognitive information from other cortical areas to plan and execute movements, enabling various gait patterns in response to environmental changes. This includes switching from automatic to controlled gait and learning new walking strategies through networks with the basal nuclei, cerebellum, and limbic structures (Takakusaki, 2023). Notably, fear of falling can influence the perception of balance, potentially exacerbating the risk of falls.

Despite improvements in basic health conditions, physical and mental conditioning, and disease prevention, ensuring healthy aging in the growing senior population remains a significant challenge (Gonçalves et al., 2014). This challenge hinges on three components: a low probability of developing diseases, minimal deficiencies in cognitive or physical-functional capacity, and active engagement with life (Araujo Nunes Marandini et al., 2017; Jantunen et al., 2017). Beyond the musculoskeletal system, impairments in neurosensory-motor integration and audiovisual temporal processing, processes crucial for simultaneity and temporal order perception, can further contribute to fall risk in older adults (Bilodeau-Mercure et al., 2015; Basharat et al., 2019).

Studies like Bueno et al. (2019) highlight the importance of neural or psychogenic factors in triggering neuromotor patterns predictive of falls, emphasizing the crucial role of individual response time to balance disturbances in fall prevention (Gélat and Chapus, 2015). Increased association time for adjustments due to these changes can significantly increase fall risk (Araujo Nunes Marandini et al., 2017; Smith and Fisher, 2018).

Considering these origins of motor control, investigating interventions with a neural focus becomes crucial. Transcranial stimulation, an emerging intervention, can modulate neural activity and regulate cortical function (Bilodeau-Mercure et al., 2015; Gélat and Chapus, 2015; Smith and Fisher, 2018; Basharat et al., 2019; Bueno et al., 2019; Li et al., 2019), potentially enhancing neuroplasticity (Gélat and Chapus, 2015; Choy et al., 2018; Basharat et al., 2019; Bueno et al., 2019; Zhang et al., 2019; Zheng et al., 2019).

Transcranial direct current stimulation (tDCS) is a non-invasive neuromodulation tool that alters spontaneous brain activity and excitability through subliminal modulation of neuronal membranes. The mechanism of action involves applying a continuous current through electrodes, flowing from the anodic to the cathodic electrode. This current passes through the skull and affects underlying brain regions, modifying the electrical potential of neuronal membranes and altering the likelihood of neurons firing. At the anode, the current tends to depolarize the membrane, facilitating neuronal excitability, while at the cathode, it can hyperpolarize and inhibit neuronal activity. Thus, tDCS can alter patterns of neural activity and produce therapeutic effects (Stagg et al., 2018).

Neuromodulation via transcranial stimulation is commonly used in treating patients with cerebrovascular and brain accidents, successfully improving residual symptoms like language (Valero-Cabré et al., 2019), dysphagia (Làdavas et al., 2015), and the manual dexterity of these patients (Alix-Fages et al., 2020). It is also cited as a recommended technique for reducing motor symptoms in older adults patients diagnosed with Parkinson’s Disease (Kamp et al., 2019).

Transcranial direct current stimulation (tDCS) has shown promise in treating falls and frailty syndrome in the older adults. tDCS is being studied in the context of rehabilitation and has been associated with exponential growth (Barboza et al., 2023). It is currently understood that tDCS can alter and strengthen synaptic activity and promote neuroplasticity, making it a valuable tool in the framework of rehabilitation (Simis et al., 2021). Studies have shown that tDCS can improve true recognition and reduce false memories in healthy older people, suggesting that it can enhance cognitive functioning (Meléndez et al., 2021). Additionally, tDCS has been found to enhance immediate memory, learning potential, and working memory in healthy older adults (Hampstead et al., 2022). These findings indicate that tDCS can be used as a non-invasive and safe method to enhance cognitive processes in the older adults, potentially reducing the risk of falls and improving frailty syndrome (Satorres et al., 2022).

However, using tDCS for fall prevention in older adults faces several challenges. Methodological gaps limit its current translational potential. While tDCS has shown promise in improving postural control and balance in both younger and older adults, results remain inconclusive. Lack of a precise understanding of tDCS mechanisms and the need for better prediction of individual response to stimulation pose major scientific challenges. Additionally, ethical issues of safety, character, justice, and autonomy must be carefully considered to protect individuals and groups in society.

While transcranial stimulation has demonstrated promising results in improving symptoms of neurological pathologies, its efficacy in directly addressing fear of falling and neuromechanical variables predictive of falls in older adults, a major risk factor for falls, remains underexplored. To address this crucial gap in knowledge, this study aims to assess the effects of non-invasive transcranial stimulation (tDCS) on fear of falling, alongside biomechanical gait and balance variables, and neuromechanical motor reaction time variables in this population. By examining these multifaceted outcomes, we hope to illuminate the potential of tDCS for mitigating fear of falling and, consequently, reducing fall risk in older adults.

## 2 Methods

### 2.1 Study design

The study is a systematic review conducted in accordance with the items of the Cochrane Handbook for Systematic Reviews of Interventions. The protocol for this review was registered in the International Prospective Register of Systematic Reviews (PROSPERO) to obtain the identification of ongoing research (ID: 222429).

The study design was made according to the following PICO strategy (Eriksen and Frandsen, 2018): adults aged 60 or older (Population), interventions by non-invasive neuromodulation (Intervention), other interventions, whether active or passive (Comparison), risk of falling, fear of falling, factors associated with the previous ones (Outcomes). The PRISMA recommendations were followed (Moher et al., 2009).

This study seeks to answer the following question: Are there evidences that support the use of non-invasive neuromodulation as an intervention in reducing the risk of falls and fear of falling in the older adults?

### 2.2 Search strategy

Searches were conducted in electronic databases: CENTRAL, Clinical Trials, LILACS, PEDro, PubMed, Web of Science, between 13/06/2020 and 13/09/2023, using the descriptors: transcranial magnetic stimulation, transcranial direct current stimulation, noninvasive brain stimulation, older adults, accidental falls, fear of fall, combined with the Boolean operators AND and OR.

A manual search of citation tracking and reference lists of articles was used to identify other eligible articles. To optimize the search strategy and find all variations of the primary terms, truncation operators and adjustments of the descriptors to the controlled vocabulary MeSH and DECs were used (Table 1).

**Table 1:** Strategies of research in eletronic databases.

Database	Research method
Central	(transcranial magnetic stimulation AND “elderly with accidental falls”) (transcranial direct current stimulation AND “elderly with accidental falls”) (noninvasive brain stimulation AND “elderly with accidental falls”) (transcranial magnetic stimulation AND “elderly with fear of falling”) (transcranial direct current stimulation AND “elderly with fear of falling”) (noninvasive brain stimulation AND “elderly with fear of falling”)
Web of Science	(transcranial magnetic stimulation AND elderly AND accidental falls) (transcranial direct current stimulation AND elderly AND accidental falls) (noninvasive brain stimulation AND elderly AND accidental falls) (transcranial magnetic stimulation AND elderly AND fear of falling) (transcranial direct current stimulation AND elderly AND fear of falling) (noninvasive brain stimulation AND elderly AND fear of falling) (transcranial magnetic stimulation AND fear of falling AND accidental falls) (transcranial direct current stimulation AND fear of falling AND accidental falls) (noninvasive brain stimulation AND fear of falling AND accidental falls)
LILACS	(transcranial magnetic stimulation AND “elderly with accidental falls”) (transcranial direct current stimulation AND “elderly with accidental falls”) (noninvasive brain stimulation AND “elderly with accidental falls”) (transcranial magnetic stimulation AND “elderly with fear of falling”) (transcranial direct current stimulation AND “elderly with fear of falling”) (noninvasive brain stimulation AND “elderly with fear of falling”)
PEDro	(transcranial magnetic stimulation with accidental falls) (transcranial direct current stimulation with accidental falls) (noninvasive brain stimulation AND with accidental falls) (transcranial magnetic stimulation with fear of falling) (transcranial direct current stimulation with fear of falling) (noninvasive brain stimulation AND with fear of falling)
PubMed	(transcranial magnetic stimulation AND “elderly with accidental falls”) (transcranial direct current stimulation AND “elderly with accidental falls”) (noninvasive brain stimulation AND “elderly with accidental falls”) (transcranial magnetic stimulation AND “elderly with fear of falling”) (transcranial direct current stimulation AND “elderly with fear of falling”) (noninvasive brain stimulation AND “elderly with fear of falling”) (transcranial magnetic stimulation OR transcranial direct current stimulation AND elderly AND fear of falling”) (noninvasive brain stimulation AND elderly AND fear of falling”) (transcranial magnetic stimulation OR transcranial direct current stimulation AND elderly AND accidental falls”) (noninvasive brain stimulation AND elderly AND accidental falls”) (transcranial magnetic stimulation OR transcranial direct current stimulation AND accidental falls OR fear of falling) (noninvasive brain stimulation AND accidental falls OR fear of falling)

Source: Developed by the authors.

### 2.3 Eligibility criteria

To prepare the review, we initially considered all indexed randomized controlled clinical trials, without restrictions on language or publication date. However, to focus on the specific effects of non-invasive neuromodulation on healthy older adults, we ultimately limited our selection to studies involving participants without reported neurological pathologies. These studies investigated the use of neuromodulation as a primary intervention for reducing fear of falling and risk of falls in this population.

Articles related to case studies, systematic reviews, observational studies, experimental studies, or those deviating from the topic were excluded. Protocols that have not yet been completed were set aside for presentation in upcoming research results, within the theme of the review.

The screening of articles was carried out by two reviewers independently, starting with reading the title and abstract, retaining articles that met the eligibility criteria and excluding those that did not, followed by confirming eligibility through reading the full text of the article. The total number of articles found in all databases was analyzed, followed by counting and excluding duplicates, using Mendeley Desktop version 1.19.4 as support.

**Figure 1:**
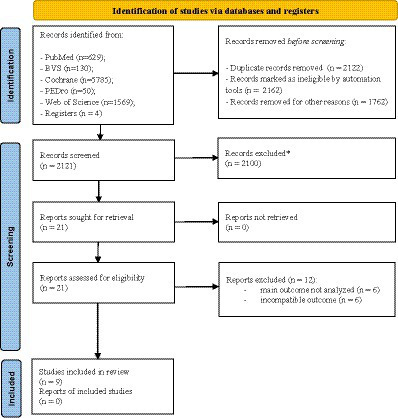
Process of selection. * excluded from the review for not meeting eligibility criteria or being irrelevant to the topic.

A comprehensive search identified 8,168 documents. Following deduplication (2,122), automated screening (3,924), title/abstract review (2,100), and full-text review (Basharat et al., 2019), 9 studies remained eligible for qualitative synthesis. Exclusion reasons for the remaining 2,100 articles were: Target data not reported (*n* = 432), focused on other outcomes (*n* = 398), investigated different exposures (*n* = 124), conducted with older adults with neurological conditions (Parkinson’s disease: *n* = 94, stroke: *n* = 475, Alzheimer’s disease: *n* = 347, peripheral neuropathy: *n* = 128), or were conference abstracts (*n* = 102). These exclusions yielded a final sample of 9 studies for qualitative synthesis using thematic analysis. Figure 1 shows the search and screening phases of the studies.

The risk of bias was assessed using the PEDro scale. The PEDro assessment scale is a tool used to evaluate the methodological quality of clinical trials in physical therapy and rehabilitation. It consists of 10 items that assess various aspects of trial quality, such as randomization, blinding, and statistical analysis. Each item is scored as either present or absent, and the scores are summed to give a total PEDro score ranging from 0 to 10. The scale has been widely used in systematic reviews and is used to rank search results in the Physiotherapy Evidence Database (PEDro). However, there has been debate about the construct validity of the PEDro scale, with some studies questioning its ability to accurately assess trial quality. Despite these concerns, the PEDro scale continues to be used as a tool for evaluating the methodological quality of clinical trials in physical therapy and rehabilitation.

The assessment was carried out independently by two reviewers (G.A.S.B. and A. D. B.), and in cases of discrepancy, the differing items were reviewed and discussed by a third evaluator to reach a consensus on the score.

Each criterion is scored based on its presence or absence in the evaluated article, and the first item called specified eligibility criteria does not contribute to the total score. The final score is obtained by summing all positive responses. Articles were considered high quality and with low risk of bias with a score of 6 or higher; thus, articles with a score below 6 were considered low quality and with a high risk of bias.

### 2.4 Data extraction and analysis

Relevant data (first author’s name, year, sample size, age, gender, description of fall risk, fall history, fear of falling, type of outcome, data collection tool, description of interventions, and result) were extracted from each included article, checked to avoid any inaccuracies or omissions, and transferred to a spreadsheet in Microsoft Office Excel. At this stage of the review, the Rayyan application, developed by the Qatar Computing Research Institute (QCRI) (Ouzzani et al., 2016), was used as an auxiliary tool for archiving, organizing, and selecting articles.

## 3 Results

### 3.1 Characteristics of the included studies

The included studies evaluated 211 Old person of both genders (*M* = 116; *F* = 95). The minimum and maximum age ranged between 65 and 85 years. The sample size of the included studies, considering all groups, was 12–57 participants. The other characteristics of the studies (details of the intervention, comparator, outcome measures, evaluation, conclusion, methodological quality of clinical trials, and financial support) are presented in Table 2.

**Table 2:** Include studies (*n* = 9) continues on the next page.

Author, year	Participants	Age	Duration	Intervention	Control	Adverse Events	Avaliation	Results	Financial aid
Ehsani (2017) Ehsani et al. (2017) Country of origin: Iran	Sex (M/F) *n* = 14/16 Intervention group: 14 older adults people (with low risk of falling and without fear of falling) Group control: 15 older adults people (with low risk of falling and without fear of falling) Sample characteristics: healthy older adults, without a history of any neurological disease (e.g., Parkinson’s disease, Alzheimer’s disease, etc.), brain surgery, brain tumor, neuropathy, visual or hearing problems, dizziness, vestibular system disorders, severe postural problems.	Intervention group: Mean: 66.08 ± 6.33 Control group: Mean: 65.50 ± 6.14	2 sessions	Stimulation type Transcranial anodal direct current stimulation (a-tDCS) Electrode Position and Size Cerebellum (1 cm below the beginning of the occipital bone) Electrode (5 × 5 cm^2^) Intensity 1.5 mA 20 min	tDCS SHAM	Intervention group: N/A Control group: N/A	Biodex balance system (BBS) Berg balance scale	↑Static postural balance ↑Dynamic postural balance ↑Motor reaction time ↔ Cerebellar tDCS and motor cortex tDCS	Does not have information
Baharlouei (2020) Baharlouei et al. (2020) Country of origin: Iran	Sex (M/F) ***n*** = 16/1 Intervention group. Older adults with low record of falling and fear of falling Control group: Older adults with low record of falling and fear of falling Sample characteristics: healthy older adults; cognitive level assessment not mentioned.	Intervention group: Mean: 67,81 ± 6,24 Control Group: Mean: 67,38 ± 6,54	2 Sessions	Stimulation type: Transcranial anodal direct current stimulation (a-tDCS) Position and electrode size Primary motor cortex (M1) Cerebellum (1 cm below the beginning of the occipital bone) Electrode (5 × 9 cm^2^) Intensity 2 mA 20 min	SHAM tDCS in Cerebellum SHAM tDCS in motor cortex	Intervention group: N/A Control group: N/A	Displacement of the Center of pressure (CoP) using the force plate (Kistler Force Plate, 9260AA6, Kistler Instruments, Switzerland)	↑Static postural balance ↑Motor reaction time ↔ Cerebellar tDCS and motor cortex tDCS	Yes
Sayig-Keren (2022) Sayig-Keren et al. (2023) Country of origin: Israel	Sex (M/F) *N* = 11/9 Intervention group: Older adults pacientes between 65 and 85 years, capable of walking at least 6 meters without any acute disease and preserved cognitive ability. Control group: Older adults pacientes between 65 and 85 years, capable of walking at least 6 meters without any acute disease and preserved cognitive ability. Sample characteristic: healthy older adults, without cognitive impairment (score greater than 20, assessed by Montreal Cognitive Assessment)	Intervention group: Mean: 72.6 ± 5.0 Control group: Mean: 72.6 ± 5.0	3 sessions	Type of stimulation Transcranial anodal direct current stimulation (a-tDCS) Transcranial direct current stimulation (tDCS) Position and size of electrode (for tACS and sham: F3, P3 and Cz; for tDCS: F3, AF4, FC5, FC1) Electrode (π cm^2^) intensity 1,500 mA for tDCS 20 min 6 Hz for tACS	tDCS SHAM tACS SHAM	Intervation group: N/A Control group: N/A	Opal, APDM, United States; Zeno mat, PKMAS software, Stroop Color and Word Test United States; Symbol digit modalities test BMI CCI MoCA Symbol digit modalities test Stroop color and word test interference score	tACS ↑ Dual-task cost for gait speed ↔ Single task gait	Yes
Mozafaripour, (2023) Mozafaripour et al. (2023) Country of origin: Iran	Sex (M/F) *n* = 0/30 Intervention group: 15 older adults woman who scored five or higher on the basis of the fall risk assessment scale Control group: 15 older adults woman who scored five or higher on the basis of the fall risk assessment scale Sample characteristics: healthy older adults; cognitive level assessment not mentioned.	Intervention group: Mean: 52.46 ± 6.00 Control group: Mean: 57.61 ± 5.86	12 sessions	Stimulation type Transcranial anodal direct current stimulation (a-tDCS) Position and electrode size active electrode (anode) was placed 1 cm below inion (Iz) to target the cerebellum, and the returning (cathode) electrode was placed over the right buccinator muscle. Electrode (5 × 7 cm^2^) Intensity 2 mA 15 min	SHAM tDCS in cerebellum Imaginary motor training	Intervention group: N/A Control group: N/A	Y balance testing BESS	↑ Static postural balance ↑Dynamic balance ↔ Balance index	No
Yosephi, (2018) Yosephi et al. (2018) Country of origin: Iran	Sex (M/F) *n* = 28/29 Intervention group: 34 older adults people (at high risk of falling and afraid of falling) Group control: 23 older adults people (at high risk of falling and afraid of falling) Sample characteristics: healthy older adults, without symptoms of amnesia and depression; memory disorders with scores below 21 on the Mini Mental State Examination	Intervention group: Mean: 66,07 ± 4,37 Control group Mean: 66,50 ± 4,24	6 Sessions	Stimulation Type Transcranial anodal direct current stimulation (a-tDCS) Electrode position and size Primary Motor Cortex (M1) Cerebellum (1 cm below the beginning of the occipital bone) Electrode (5 × 7 cm^2^) Intensity 2 mA 20 min	tDCS simulation with postural training Postural training alone	Intervention group 1 pre-intervention hospitalization 2 withdrawals due to work problems Group control: AT.	Biodex balance system (BBS) Berg balance scale	↑Static postural balance - cerebellar tDCS ↑Dynamic postural balance - tDCS motor cortex ↑Motor reaction time - tDCS motor cortex	Does not have information
On-Yee Lo (2019) Lo et al. (2023) Country of origin: United States	Sex (M/F) *n* = 1/5 Intervention group: Men and women aged 65 years and above, who were referred to PT for gait and balance training due to recurrent falls or high risk of falling at the outpatient geriatric physical therapy clinic within the senior healthcare organization. Control group: Men and women aged 65 years and above, who were referred to PT for gait and balance training due to recurrent falls or high risk of falling at the outpatient geriatric physical therapy clinic within the senior healthcare organization. Sample characteristic: healthy older adults, without cognitive impairment (score greater than 18, assessed by montreal cognitive assessment)	Intervention group: Mean: 52.46 ± 6.00 Control group: Mean: 57.61 ± 5.86	10 sessions	Stimulation type tDCS Position and Size of the electrode left dorsal lateral prefrontal cortex (DLPFC) 6 electrodes (size not informed) Intensity <4 mA 20 min	tDCS sham stimulation and physiotherapy	Intervention group N/A Control group N/A	TUG; FES-I; MOCA; TMT; Berg balance Scale; TMTadj	↑BBS ↔ on normal and dual task gait speed, TUG, FES-I, MoCA, and TMT adjusted	Yes
Schneider, (2021) Schneider et al. (2021) Country of origin: Israel	Sex (M/F) *n* = 5/20 Intervention group: N/A Intervention group walking N/A Control group N/A Sample characteristic: healthy older adults, with global rating of 0.5 on the Clinical Dementia Rating scale	Intervention group Mean: 73.9 ± 5.2 Control group N/A	3 sessions	Stimulation type Transcranial direct current stimulation (tDCS) Sit or walking realising motor-cognitive task Position and size of electrode Targeting both the primary motor cortex (M1) and the left dorsolateral prefrontal cortex (lDLPFC) Electrode (π cm^2^) Intensity The target En-field was set to +0.25 V/m over each designated ROI, and 0 V/m over the remaining regions Based on 10–20 system 20 min	sham simulation applied during the performance of the motor-cognitive walking task	Intervention group N/A Control group N/A	Opal, APDM, United States; Zeno mat, PKMAS software, Stroop Color and Word Test United States; Symbol digit modalities test	↓ Dual-task cost to gait speed for tDCS + walking (p = 0.004) ↔ Stroop performance. Sway	Yes
Kaminski (2017) Kaminski et al. (2017) Country of origin: Germany	Sex (M/F) *n* = 13/17 Intervention group: 15 older adults with low risk of falling. Control group: 15 older adults with low risk of falling. Sample characteristics: healthy older adults; underwent a detailed neurological examination; no signs of cognitive impairment, as measured by the Mini-Mental State Examination.	Intervention group: mean: 67,7 ± 6 years Control group: mean: 66,9 ± 3 years	2 sessions	Stimulation duration Transcranial anodal direct current stimulation (a-tDCS) Position and electrode size Primary Motor Cortex (M1) Electrode (5 × 5 cm^2^) Intensity 1 mA 20 min	tDCS SHAM	Intervention group: N/A Control group: N/A	Dynamic balancing task on a force platform (LaFayette Instruments, model 16,030, Lfayette, IN, United States) Attention rating scale Analogic visual scale	↑ Learning ↔ Static postural balance ↔ Speed and acceleration of the Center of Gravit	Yes
Smith (2018) Smith and Fisher (2018) Country of origin: United States	Sex (M/F) *n* = 7/18 Intervention group: 12 older adults (5 fallers and 7 non-fallers): Control group: 13 young adults Sample characteristics: healthy older adults, independent in activities of daily living and ambulation; cognitive level assessment not mentioned.	Intervention Group Mean: 72,42 ± 8,16 Control group: Mean: 25,75 ± 2,09	1 session	Stimulation Type Transcranial magnetic stimulation Position and electrode size Contralateral hemisphere to the EMG measurement (i.e., left if the dominant limb was the right) Double cone coil of 110 mm Intensity Pulses every 5–10 s 20 min	Young adults without intervention	Intervention group: An old man without history of falling that does not complete the data collection due to fatigue Control group: A young woman’s postural anticipation data were not recorded due to equipment failure	BESTest; Timed up and go test Gait – 10 meter walk test All three tests were evaluated based on Anticipatory Postural Adjustments, using surface Electromyography on the muscles: - External Oblique - Paravertebral - Gluteus Medius Biodex Balance System (BBS) Berg Balance Scale	↑ spatial organization of the motor cortex ↔ temporal organization of anticipatory postural adjustments during gait. ↔ Motor reaction time ↑ Marching speed of fallers	Yes

↔ No significantly difference; ↑ significantly higher; ↓ significantly lower; < significantly, but not expressively; N/A, not applicable.

The overall therapeutic intervention period ranged from 1 to 6 sessions. The frequency of the intervention and control groups varied from 1 to 2 times per week. The duration of the intervention (session) ranged from 20 to 40 min, combined with other modalities, and 20 min for tDCS exclusively. The dosage of transcranial direct current stimulation varied from 1 to 2 mA.

### 3.2 Ongoing studies

In our search, we found 4 ongoing studies at https://clinicaltrials.gov/ (Table 3). Table 4 shows that the methodological quality score of the included studies ranged from 6 to 9 points on the PEDro scale. Of the items scored on the PEDro scale, none of the studies scored item 6 (blind therapists). In contrast, all studies scored on item 9 (data analysis with treatment or with intent to treat) and 10 (comparisons between groups). Regarding the other items, each study had its score if it met the criteria for each of the items. Therefore, only 9 studies make up this review, as they are classified with high methodological quality (PEDro ≥6).

**Table 3:** Ongoing studies.

**Year**	**Title**	**Participants**	**Intervention**	**Control**	**Outcome**
Ing-Shiou (2015)	Enhancement of Posture Training Effectiveness with Error-enhancing Feedback and Cerebellar Stimulation	Adults aged 60 and over	Transcranial direct current stimulation	Simulated transcranial cerebellar stimulation No intervention: feedback that improves the error	Primary: graphical theoretical analysis of the EEG functional network Secondary: center of pressure direction analysis
Manor (2020)	Personalized Brain Activity Modulation to Improve Balance and Cognition in Older adults Fallers	Adults between 65 and 85	Transcranial direct current stimulation	Simulated stimulation	Primary: dual task cost for walking speed; dual task cost for standing postural sway speed; short physical performance battery (SPPB); trail making test B - A Secondary: dual-task cost for serial subtraction performance; dual task cost to overcome time variability; dual task cost of walking dual support time; dual task cost for elliptical area of standing postural sway; timed up-and-go (TUG); falls efficacy scale (FES-I); 5-day accelerometry-based habitual physical activity; montreal cognitive assessment (MoCA); Digit span; WAIS-IV coding test; phonemic and category fluency test; Hopkins verbal learning test
Padala (2020)	Neuromodulation for Exercise Adherence in Older Veterans	Adults aged 60 and over	Transcranial magnetic stimulation	Simulated stimulation Non-intervention	Primary: Change in adherence measured by number of minutes; conners continuous performance test; change in adherence measured by number of sessions Secondary: walking speed; timed up and go
Qi et al. (2019)	The effects of non-invasive transcranial direct current stimulation (TDCS) on posture over stable and unstable surfaces in healthy older people: a randomised double-blind sham-controlled study	Adults (63.13 ± 0.97 anos)	Transcranial direct current stimulation	Simulated stimulation	Primary: balance control during single and dual task conditions while maintaining stable and unstable surfaces

**Table 4:** PEDro scale.

Author, year	2 Randomized alocation	3 Blind alocation	4 Comparability of line base	5 Blind Participants	6 Blind Therapist	7 Blind assessors	8 < 15% dropout rate	9 Intention-to-treat analysis	10 Comparison between groups	11 Point estimates and variability	Total
Ehsani (2017) Ehsani et al. (2017)	1	1	1	1	0	1	1	1	1	1	9
Baharlouei (2020) Baharlouei et al. (2020)	1	1	1	1	0	1	1	1	1	1	9
Sayig-Keren (2022) Sayig-Keren et al. (2023)	1	1	1	1	1	0	1	1	1	1	9
Mozafaripour (2023) Mozafaripour et al. (2023)	1	1	1	1	1	0	1	1	1	1	9
Yosephi (2018) Yosephi et al. (2018)	1	1	1	1	0	1	1	1	1	0	8
On-Yee Lo (2019) Lo et al. (2023)	1	1	1	1	0	1	0	1	1	1	8
Schneider (2023) Schneider et al. (2021)	1	1	1	1	1	0	0	1	1	1	8
Kaminski (2017) Kaminski et al. (2017)	1	1	1	1	0	0	1	1	1	0	7
Smith (2018) Smith and Fisher (2018)	0	0	1	1	0	0	1	1	1	1	6

### 3.3 Summary of main results

The review focused on evaluating the efficacy of non-invasive neuromodulation (tDCS, tMS and tACS) versus control (S-tDCS, any other approach to reduce the risk of falls in the older adults). Nine trials with a total of 240 (95 males; 145 females) participants were included. Four studies (Ouzzani et al., 2016; Ehsani et al., 2017; Kaminski et al., 2017; Baharlouei et al., 2020) with 98 (35 males; 65 females) participants addressed the outcome measure for fall risk: investigating the effect of tDCS versus control on motor reaction time, static and dynamic postural balance (center of pressure displacement).

Only two studies (Kaminski et al., 2017; Schneider et al., 2021), showed no evidence of post-intervention effect on static and dynamic balance (displacement and speed of the center of pressure). The authors attribute the lack of positive findings to tDCS, the applied methodology, where prior training of the motor activity to be performed promoted learning, thus masking possible positive results.

Effects of non-invasive neuromodulation on functional activities were the outcome of a single article (Smith and Fisher, 2018), which was also the only work that used Transcranial Magnetic Stimulation as an intervention. We found evidence of an effect post-intervention on fall protective mechanisms (increased walking speed in older adults fallers), even though there is a fragility of the intervention period (one session). However, when looking at the work, it is not possible to clearly discern the absence of evidence on motor reaction time and consequent temporal organization of anticipatory postural adjustments during walking, since the intervention group is a mix of fallers and non-fallers, with the intra-group intervention comparison being vulnerable to statistical error considering a sample of 12 older adults (divided into five fallers and seven non-fallers).

One study showed (Ouzzani et al., 2016; Mozafaripour et al., 2023) that were significant improvement in both balance tests in the intervention group after intervention compared to the control group. Both static and dynamic balance improved significantly from the baseline values only in the intervention group and not in the control group. No direct pre- and post-intervention risk assessments were reported.

No adverse events directed at the intervention were reported, and the dropout rate was low, being present in only three studies (Smith and Fisher, 2018; Yosephi et al., 2018; Lo et al., 2023).

## 4 Discussion

### 4.1 Total completeness and applicability of evidence

The results of this review seem to be quite promising for a short-term intervention and producing satisfactory results for reducing the risk of falls in the older adults. However, there are some factors that produce uncertainty for a satisfactory conclusion. These are: all studies included older adults of both sexes, without interpreting the influence of sex on the data; all studies characterize the fall risk of the older adults, with two reporting the history of falls (Smith and Fisher, 2018; Baharlouei et al., 2020) and two reporting the fear of falling (Moher et al., 2009; Ehsani et al., 2017; Eriksen and Frandsen, 2018; Yosephi et al., 2018; Kamp et al., 2019; Baharlouei et al., 2020), but none of them show these data as post-intervention outcomes.

### 4.2 Heterogeneity in intervention duration

Thus, the results may have limited applicability for direct outcomes to fall risk, in addition to evidence regarding the difference or not in applicability between sexes, fallers and non-fallers, as well as older adults with low and high fear of falling. A standardization regarding electrode size, positioning, intensity, and session duration was found in the studies.

Currently, there is not enough high-quality evidence to draw conclusions about the benefits or harms of TDCS. However, as there is no evidence of serious adverse effects and it can be easily administered, further research on tDCS is justified.

Regarding the comparable dropout rate between groups, it should not be assumed that the small number of dropouts in the included trials would be transferred to daily clinical practice.

### 4.3 Potential biases in the review process

The methodological rigor of the Systematic Reviews and Meta-Analyses: The PRISMA Statement minimizes bias in the process of conducting systematic reviews (Moher et al., 2009). However, some aspects of this review are open to bias, even though the studies were reviewed by two authors and a third was consulted when there was disagreement, and a considerable number of databases were used as a strategy, this does not guarantee that the risk of bias in the search is absent.

### 4.4 Agreements and disagreements with other studies or reviews

To the best of these authors’ knowledge, there is no other systematic review with the directed outcome. Other systematic reviews sought to evidence the effects of non-invasive neuromodulation on motor aspects in the older adults but directed at patients with motor disorders due to neurological conditions (Summers et al., 2016; Roncero et al., 2020; Shoaib et al., 2023).

The evidence from tDCS, the articles point out that from two to six sessions, positive effects are observed on postural balance and indirectly on the fall risk of the older adults (Ehsani et al., 2017; Yosephi et al., 2018; Baharlouei et al., 2020; Schneider et al., 2021; Lo et al., 2023; Mozafaripour et al., 2023; Sayig-Keren et al., 2023). These are satisfactory evidence, as interventions directed at the musculoskeletal system indicate that 12 to 20 weeks of balance training are needed to reduce the fear of falling and improve dynamic balance indices in older adults at high risk of falling (Judge et al., 1993; Lord and Castell, 1994; Gusi et al., 2012).

### 4.5 Conclusions and future research directions

Although we did not find results directly related to the risk of falls and fear of falling in the older adults through neuromodulation, it should be mentioned that it is a topic recently targeted by researchers in the scientific community, as only in 2023 we found three clinical trial registrations for these outcomes.

Another important point to consider is the attention that clinical trials give to the cognitive aspect of participants. Of the nine clinical trials included in this review, six investigated the cognitive state of the sample, excluding participants who showed any decline (Ehsani et al., 2017; Kaminski et al., 2017; Yosephi et al., 2018; Schneider et al., 2021; Lo et al., 2023; Sayig-Keren et al., 2023).

Falls and cognition share neurobiological mechanisms that contribute to fall risk in older adults. Cognitive impairment, including deficits in attention, executive function, and information processing speed, has been found to increase the risk of falls (Montero-Odasso and Camicioli, 2020; Le Floch et al., 2021; Edwards et al., 2023). Neuroimaging studies have revealed changes in brain networks, such as the fronto-parietal and subcortical networks, that are associated with both physical and cognitive function and are implicated in age-related falls (Rosso et al., 2020; Zhang et al., 2020). Specifically, alterations in the structural integrity, vascular characteristics, and functional activity of the central nervous system have been identified as potential contributors to falls. These findings suggest that age-related neurobiological changes in cognition and the central nervous system may underlie the increased risk of falls in older adults. Further research is needed to better understand the specific mechanisms and develop targeted interventions for fall prevention in this population. Neuromodulation may act on these shared mechanisms by targeting the CNS changes associated with both cognitive decline and fall risk. However, further research is needed to understand the specific neurophysiological outcomes of neuromodulation in relation to cognition and falls.

The study of falls in the older adults is advancing in the scientific community, with a focus on prevention and prediction, and in yet-to-be-explored areas, such as the biomarkers that determine this event.

Neurodegeneration biomarkers, such as MRI brain atrophy and [18F]FDG-PET hypometabolism, can be used to predict the risk of falls. Combined atrophy and hypometabolism, as assessed by MRI and [18F]FDG-PET, have been found to predict progression over 1 year in patients suspected of neurodegenerative disease (Olson et al., 2021). Neurophysiological techniques that evaluate synaptic function and brain connectivity can also serve as biomarkers for screening the risk of falls in individuals with mild cognitive impairment (MCI). These techniques, when combined with artificial intelligence methods, have shown promising results in identifying prodromal-to-dementia MCI subjects (Gramkow et al., 2020). Additionally, wearable technologies, such as inertial measurement units (IMUs), have been used to continuously monitor aspects of gait, balance, and other health-related factors known to be associated with falls. Measures of gait speed, step length, and entropy have been found to correlate with fall risk, and machine learning methods can distinguish between falls (Rossini et al., 2020).

Non-invasive neuromodulation, as highlighted in this systematic review, is being studied in relation to falls, but its specific relationship with neurodegeneration biomarkers and neurophysiological measures is not mentioned in the studies provided. Future studies should investigate the specific relationship between non-invasive neuromodulation and neurodegeneration biomarkers and neurophysiological measures. This would help to better understand how non-invasive neuromodulation works to prevent falls in the older adults and could lead to the development of new treatment strategies.

In general, this review contributes to the indication of the size and positioning of the electrodes, as well as the intensity and duration of a session, but leaving open the need for further studies to elucidate the positive effects and especially the long-term effects.

## Data availability statement

The original contributions presented in the study are included in the article, further inquiries can be directed to the corresponding author.

## Author contributions

GB: Conceptualization, Data-curation, Formal analysis, Funding acquisition, Investigation, Methodology, Project administration, Resources, Software, Supervision, Validation, Visualization, Writing – original draft, Writing – review & editing. AB: Data curation, Writing – review & editing. LC: Data curation, Methodology, Writing – original draft. AM: Formal analysis, Supervision, Writing – review & editing. RM: Formal analysis, Supervision, Writing – review & editing. RE: Investigation, Project administration, Resources, Visualization, Writing – original draft, Writing – review & editing. MS: Funding acquisition, Methodology, Resources, Supervision, Visualization, Writing – original draft, Writing – review & editing. SF: Funding acquisition, Methodology, Resources, Supervision, Visualization, Writing – original draft, Writing – review & editing.
